# Threshold studies in food allergy

**DOI:** 10.1186/2045-7022-1-S1-S19

**Published:** 2011-08-12

**Authors:** Barbara Ballmer-Weber

**Affiliations:** 1 Leitende Ärztin Allergiestation und Epikutanlabor, Dermatologische Klinik, Universitätsspital Zürich, Zürich, Switzerland

## 

Thresholds constitute a critical piece of information in assessing the risk from allergenic foods at both the individual and population levels. Knowledge of the minimum dose that can elicit a reaction is of great interest to all food allergy stakeholders. Threshold is defined as a limit below which a stimulus causes no reaction. Associated with the concept of threshold are the toxicological terms of No Observed Adverse Effect Level (NOAEL) defined as the highest dose of a substance observed in a study not to produce any adverse effect and Lowest Observed Adverse Effect Levels (LOAEL) defined as the lowest dose that is observed to produce an adverse effect. Many factors can affect threshold values. In particular it has been demonstrated that the fat content of the matrix has an impact on the amount of allergens leading to an allergic reaction. Study protocols for threshold determination include low dose challenges starting at very small doses, preferably below published thresholds to get informations on NOEALs. Most importantly, the cumulative challenge dose has to be high enough to confirm food allergy within the study population. Active and placebo provocations are performed on two different days. Challenges are usually discontinued after the dose leading to the first objective allergic symptoms. Most reliable data for threshold levels have been obtained to date in peanut allergic subjects. An ED10 (the dose predicted to provoke a reaction in 10% of the peanut-allergic population) was recently calculated to be 12.3 mg of whole peanut taking into account data of 450 challenged subjects. In the EuroPrevall project, the first subjective symptoms occurred for all tested foods between 3 and 30ug protein. LOAELs based on objective symptoms were observed for celeriac at 30ug, followed by hazelnut and milk at 300ug.

